# Actigraphic Recording of Manic Symptoms Induced by Methylphenidate

**DOI:** 10.1155/2009/286430

**Published:** 2009-10-15

**Authors:** Tuuli Lahti, Sami Leppämäki, Pekka Tani, Timo Partonen

**Affiliations:** ^1^Department of Mental Health and Substance abuse Services, National Institute for Health and Welfare, 00271 Helsinki, Finland; ^2^Department of Psychiatry, Helsinki University Central Hospital, 00290 Helsinki, Finland

## Abstract

*Objective*. Attention-deficit hyperactivity disorder (ADHD) is a developmental disorder characterized by a long-standing pattern of impulsive behavior, hyperkinesia, and inattention. Psychostimulants, for example, methylphenidate, are the treatment of choice for ADHD both in children, adolescents, and adults. *Method*. The effect of methylphenidate on sleep structure is not well known. We studied the effect of long-acting methylphenidate on sleep in adult ADHD patients, in a naturalistic treatment setting, using actigraphic and polysomnographic recordings. *Results*. One of our patients experienced manic episode after starting methylphenidate. A wrist-worn accelerometer recording demonstrated a decrease in the duration and quality of sleep. After discontinuation of methylphenidate treatment, the patient's symptoms subsided and there was no need for hospital admission. Actigraphic recording showed a decrease in the amount and quality of the patient's sleep as triggering factor for the manic symptoms. *Conclusions*. Disruptions of the sleep-wake cycle are probably important etiologic factors in mood disorders, especially bipolar disorder. The changes in length and quality of sleep described in this case report bear close resemblance to those of patients with a manic episode, although these symptoms were induced by methylphenidate.

## 1. Introduction

Attention-deficit hyperactivity disorder (ADHD) is a developmental disorder characterized by impulsive behavior, hyperkinesia and inattention. Recent studies suggest that there is a range of abnormalities in neurotransmission in ADHD patients [1]. Psychiatric comorbidity is common in adult ADHD patients [2]. In addition, other developmental disorders, for example, dyslexia, and sleep problems are frequent in patients with ADHD [3].

Severe ADHD symptoms are often treated with medication. Psychostimulants are effective in all symptom domains of ADHD. Stimulants such as dextroamphetamine and methylphenidate are used in the treatment of ADHD both in children and adults. A common adverse effect of methylphenidate is a sleep disturbance. In children, even short-acting methylphenidate thrice daily increases sleep onset latency [4]. However, in clinical experience, adult ADHD patients with prominently hyperactive symptoms in particular appear to sleep better after a stimulant has been started. This in mind, we assessed the night-time sleep and the daily rest-activity cycles in adult ADHD patients before, during and after long-acting methylphenidate treatment (Concerta).

One of the study participants, a 40-year-old man with no prior history of bipolar disorder, experienced a manic episode after the treatment with methylphenidate was started. His switch from euthymia into mania was recorded with a wrist-worn accelerometer. We present herein, for the first time, actigraphic recording data on a switch from euthymia into mania induced by methylphenidate.

## 2. Methods

The diagnosis of ADHD was assessed with the Conners' Adult ADHD Diagnostic Interview for DSM-IV [5] using multiple sources of information. Mental disorders were excluded using SCID-I and SCID-II-interviews [6, 7]. A comprehensive neuropsychological test battery was included in the diagnostic process. At baseline, all the patients had a careful physical examination including laboratory tests and a urine screen. The study protocol was approved by the ethics committee of the Helsinki University Central Hospital, Helsinki, Finland. All the patients volunteered for the study and signed an informed consent after the protocol had been fully explained and before any procedure was performed. Here, we present a case report of one of the study participants, a 40-year-old man. He was recently diagnosed as having ADHD, with no medication prior to the study. The dosage was at start one tablet (18 mg of methylphenidatehydrochloride) in the morning, and after six days of medication the daily dose was increased up to two tablets (36 mg of methylphenidatehydrochloride) in the morning. Concomitant psychotropic medication was not allowed during the study. The daily rest-activity cycles were continuously recorded with a wrist-worn accelerometer (actigraph). It gives information about the duration and quality of sleep and the level of physical activity. He wore the accelerometer on his nondominant hand all the time, except during bathing or swimming, for 16 days before starting the treatment with methylphenidate and for 11 days (18 mg for 6 days followed by 36 mg for 5 days) during the active treatment. The actigraphic variables used for the analysis included sleep efficiency (the percentage of time spent asleep whilst in bed), sleep latency (the time delay between going to bed and falling asleep), actual sleep time (the percentage of time spent in bed in a sleep), actual wake time (the percentage of actual time spent awake in bed), mean activity score (the average value of the activity counts per epoch over the assumed sleep period), movement and fragmentation index (indicates the restlessness of the sleep), number of minutes moving (the total number of minutes moving during sleep), and number of minutes immobile (the total number of minutes immobile). The data were analyzed with the software provided by the manufacturer (The Actiwatch Sleep Analysis 2001, version 1.19).

## 3. Results

The patient's response to methylphenidate was clinically rated as good. In addition to alleviation of his ADHD symptoms, his bruxism and nail-biting activities were reduced. However, he became increasingly more talkative and excited during the treatment with methylphenidate. He reported that his subjective quality of sleep was decreased, which was confirmed in the actigraphic recordings (Table 1). After doubling the methylphenidate dosage from 18 mg to 36 mg per day, he became more impulsive. He described experiencing a totally new understanding of life and some kind of bonding with the universe. He had rapid mood swings during the days. He felt very energetic and was exceptionally active. The diagnosis of a manic episode (substance-induced) was assessed, when he had an appointment (per study protocol) with one of the authors (PT). His wife was met during the appointment and confirmed the change in activity and behavior. The patient was excluded from the study and his treatment with methylphenidate stopped immediately. His manic symptoms disappeared completely during the following days, and no mood-stabilizing medication was needed. In the actigraphic recordings, the quality and the duration of sleep were reduced during the treatment with methylphenidate (Table 1). Sleep efficiency was reduced in proportion to the methylphenidate dose. Sleep latency was increased, actual sleep time was decreased, and actual wake time was increased during the treatment. All the activity variables indicated that sleep became increasingly more restless as the methylphenidate dose increased.

## 4. Conclusions

Insomnia is known to be a potential adverse effect of psychostimulants and may trigger manic episodes [8, 9]. Patients with mood disorder in particular are vulnerable to sleep disruption, and among bipolar patients with the comorbid ADHD the rate of the stimulant-associated hypomania or mania is as high as 40% [10–15]. Our patient had no prior history of depressive, hypomanic or manic episodes. During a follow-up of three years, our patient has experienced no further episodes of mood disorder, although he is still being treated with methylphenidatehydrochloride (mainly short-acting tablets, 20–25 mg/day). However, he has reported that occasionally, after the use of longer-acting methylphenidate tablets for three or more consecutive days, insomnia returns and his symptoms start to resemble those experienced during the manic episode.

Clinical experience is that disruptions in sleep often predict onset of mood episodes, both mania and depression. Here, methylphenidate disrupted sleep and induced mania-like symptoms in an adult patient with ADHD, but no underlying mood disorder. His reaction seemed to be clearly dose-related and dependent also on the use of long-acting methylphenidate.

## Figures and Tables

**Table 1:** Mean values for actigraphic parameters before and during treatment with methylphenidate.

	Daily dosage and duration of treatment with methylphenidate		
Parameter	0 mg for 16 days	18 mg for 6 days	36 mg for 5 days
Sleep efficiency (%)	80.4	69.0	67.4
Sleep latency (h : min)	0 : 4	1 : 2	1 : 1
Actual sleep time (%)	87.8	86.7	80.0
Actual wake time (%)	12.2	13.4	20.0
Mean activity score	12.1	13.5	26.5
Movement and fragmentation index	31.2	34.9	44.8
Number of minutes moving	14.1	15.7	22.3
Number of minutes immobile	86.0	84.4	77.7
